# Generalizing the Gaussian Network Model: Spanning‐Tree Thermodynamics Shows Entropy‐Driven KRAS Activation

**DOI:** 10.1002/prot.70146

**Published:** 2026-06-08

**Authors:** Fatma Senguler Ciftci, Burak Erman

**Affiliations:** ^1^ Department of Chemical and Biological Engineering Koç University Istanbul Turkey

**Keywords:** allosteric network, Kirchhoff Laplacian, KRAS, matrix‐tree theorem, spanning‐tree partition function

## Abstract

The GTPase KRAS executes a conformational switch between a GTP‐bound active state and a GDP‐bound inactive state, a process central to oncogenic signaling. However, the structural basis of this switching at the level of residue‐contact organization remains incompletely characterized by traditional binary structural models. Here, we present a statistical‐mechanical generalization of the Gaussian Network Model (GNM) by constructing spanning‐tree partition functions for residue‐contact graphs using the weighted Kirchhoff Laplacian in conjunction with the Matrix‐Tree Theorem. Within this framework, the standard GNM is recovered in the high‐temperature limit, whereas the present formulation enables a continuous Boltzmann‐weighted ensemble analysis. We compute the network free energy F, mean contact energy $\bar{E}$, heat capacity $C_{v}$, and thermodynamic entropy S across an effective temperature sweep that maps the combinatorial diversity of the contact network, thereby probing the topological landscape rather than structural melting. Differential analysis reveals that KRAS activation reflects a systematic entropy‐enthalpy compensation mechanism: the active state incurs a systematic energetic penalty $\left(\Delta \bar{E}>0\right)$ that is offset by a marked gain in conformational entropy $\left(\Delta S>0\right)$, with a free‐energy crossover occurring at kT≈2.41Å. Edge marginal inclusion probabilities, obtained via effective‐resistance theory, identify Switch I (residues 25–40) as the primary allosteric locus of nucleotide‐driven network reorganization. This approach provides a thermodynamically grounded perspective on KRAS allostery, quantitatively demonstrating how network architecture enables functional versatility through entropy‐driven conformational flexibility.

## 1 Introduction

KRAS is the most frequently mutated oncogene in human cancer: activating mutations at positions G12 and G13 appear in roughly 30% of all tumors [1]. Functioning as a molecular switch, the protein cycles between GTP‐bound active and GDP‐bound inactive conformations to regulate proliferation, survival, and differentiation through downstream effectors including RAF, PI3K, and RALGDS [2]. This clinical importance has driven intensive efforts toward direct small‐molecule inhibitors [3]. Thermodynamic and kinetic aspects of KRAS allostery and its downstream interactions have been studied by Manley and Lin [4], while Killoran and Smith [5] have characterized nucleotide cycling and effector interactions across multiple GTPases at conformational resolution. A substantial and growing body of work addresses KRAS allostery through GTP‐dependent conformational dynamics, reorganization of interaction networks, and thermodynamic analyses of binding energetics [6, 7, 8]. Integrative approaches combining Markov state modeling with allosteric network analysis have further mapped KRAS‐effector binding landscapes and shown how mutations redistribute communication pathways [9]. These studies have mapped allosteric communication through conformational sampling, mutational scanning, and network centrality analysis, yet the thermodynamic consequences of global contact‐network reorganization upon nucleotide exchange have not been assessed quantitatively. A central open question is how the global architecture of residue contacts, rather than any single interaction, collectively enables or restricts allosteric signal transmission between the nucleotide‐binding pocket and distal effector surfaces. This question is especially acute for KRAS, where global network reorganization upon nucleotide exchange may be a more fundamental determinant of functional switching than local structural rearrangements alone.

Conventional structural analyses compare active and inactive states by cataloging residue pairs that gain or lose contacts upon nucleotide exchange [10, 11]. Elastic network models extend this picture with coarse‐grained descriptions of collective motions. Still, because they are defined about a single energy minimum, they cannot capture the multiplicity of communication pathways accessible to the network [12]. Recent work has advanced the field by deriving edge centrality measures that implicitly enumerate communication pathways through the matrix‐tree theorem, revealing conserved allosteric bottlenecks in KRAS [13]. That framework, however, does not assign explicit statistical weights to individual spanning‐tree configurations and therefore cannot yield a temperature‐dependent partition function from which heat capacity, switching free energy, and entropic contributions to state transitions follow. Neither class of approach provides what is needed: a thermodynamically consistent partition function that assigns statistical weights to contact configurations and yields well‐defined free energy, entropy, and heat capacity for the network as a whole. Without such a description, the thermodynamic cost of switching between states and the relative contributions of energetic versus entropic terms cannot be assessed from static structural data. The framework introduced here addresses this gap directly by summing over the complete ensemble of contact network configurations, yielding thermodynamic quantities that are immediately comparable between the two nucleotide states.

Protein contact networks exhibit small‐world communication properties, with short path lengths that enable efficient signal propagation across the structure [14]. We represent the protein's residue‐contact network as a spanning tree, an acyclic subgraph that includes all *N* residues and connects them using exactly *N*−1 contacts. Each spanning tree constitutes a minimal allosteric backbone: the smallest subset of contacts through which a perturbation at the nucleotide‐binding pocket can reach every other residue without redundancy. The partition function enumerates all such backbones, each weighted by its Boltzmann factor, and thereby quantifies the combinatorial diversity of allosteric routes available at a given effective temperature. Earlier approaches have characterized network topology through weighted Laplacian spectra and perturbation analysis [15], or through centrality rankings and coevolutionary methods [16]. The present work extends this programme by assigning explicit Boltzmann weights to individual spanning‐tree configurations, converting the spectral description into a statistical‐mechanical ensemble from which thermodynamic potentials follow exactly.

Our formulation is grounded in algebraic graph theory [17, 18]. Each KRAS structure is represented as a weighted residue contact graph, and its spanning‐tree partition function is evaluated as a Boltzmann‐weighted sum over all connected, cycle‐free subgraphs that span the full residue set. This statistical‐mechanical description shows how allosteric signals distribute across competing pathways, with each tree configuration's weight reflecting its total interaction energy at a given effective temperature. Changes in this combinatorial diversity upon nucleotide exchange are invisible to pairwise contact analyses or single‐minimum elastic descriptions; it is precisely these changes that, we argue, define the thermodynamic distinction between the active and inactive states.

A related graph‐theoretic approach was developed by Senet and co‐workers, who defined a local entropy based on subgraph enumeration around each residue to quantify conformational heterogeneity across folded, unfolded, and intrinsically disordered states [19]. Their local entropy functions as an order parameter for protein unfolding and correlates with NMR chemical shifts. The present work takes a global perspective: the partition function sums over all connected, cycle‐free subgraphs spanning the entire residue set, yielding network‐level free energy, entropy, and heat capacity rather than residue‐level disorder measures.

The theoretical foundation of the Gaussian Network Model (GNM) derives from the theory of elastic network constraints established by Flory and Erman [20], who established the early physical basis for modeling junction fluctuations in constrained systems. The model [21] approximates residue interactions with a binary contact function: residues within a cutoff distance interact with uniform strength; those beyond do not interact at all. Computationally convenient, this replaces the continuous spectrum of interaction strengths with a present/absent dichotomy. We adopt a different approximation. A hard cutoff $d_{c}=8.0$ Å defines which residue pairs form edges in the contact graph; within this graph, each edge receives a continuous Boltzmann weight that decays smoothly with inter‐residue distance. This replaces the uniform‐strength assumption of the GNM with a heterogeneous weighting that transforms the network from a single‐minimum harmonic system into a statistical‐mechanical ensemble over all connected configurations. This transition from a purely topological representation of a continuously weighted interaction landscape is what enables the derivation of thermodynamic potentials from the elastic framework. As shown formally in § 2.8, the standard GNM is recovered as the high‐temperature limit of this ensemble, where all weights converge to unity, and the partition function reduces to the spanning‐tree count. The generalization opens access to the full thermodynamic apparatus of partition functions, free energies, and entropies, all derivable exactly from network topology without additional approximation or sampling.

Applying this framework to two crystallographic wild‐type KRAS structures, 6GOD (GTP‐analog‐bound, active) and 4OBE (GDP‐bound, inactive), we find that activation cannot be described as a purely energetic transition. Rather, it reflects entropy‐enthalpy compensation: the active state incurs a systematic energetic penalty ($\Delta \bar{E}>0$) offset by a gain in conformational entropy (ΔS>0), with the free‐energy balance crossing at a well‐defined intrinsic temperature. Switch I (residues 25–40) emerges as the primary site of nucleotide‐driven network reorganization.

## 2 Methods

The analysis proceeds in a sequence of deterministic steps from atomic coordinates to thermodynamic observables. No stochastic sampling, molecular dynamics, or normal‐mode approximation is involved at any stage. Every parameter choice is collected in Table 3 at the end of this section; the complete source code is publicly available (§ 6).

### 2.1 Structural Data

Two wild‐type KRAS crystal structures were obtained from the RCSB Protein Data Bank: 6GOD (GTP‐analog‐bound, chain A, 1.90 Å resolution, 172 resolved residues) and 4OBE (GDP‐bound, chain A, 1.60 Å resolution, 169 resolved residues). Only the C*α* atom of each residue was retained. No alignment, superposition, or renumbering was applied; residue indices match the deposited PDB numbering throughout. The two structures share identical numbering over their common range; 4OBE simply lacks Met170, Ser171, and Lys172 at the C‐terminus.

Each structure thus yields an ordered list of n residues with three‐dimensional coordinates $r_{i}=\left(x_{i},y_{i},z_{i}\right)$, where n=172 for 6GOD and n=169 for 4OBE.

### 2.2 Contact Graph and Cutoff

From these coordinates, we build a residue contact graph $G=\left(V,e\right)$, with one node per residue (∣V∣=n). An undirected edge $e=\left(i,j\right)$ is placed whenever the C*α*–C*α* distance falls below a hard cutoff:

(1) $$d_{\mathrm{ij}}\equiv \;∥r_{i}-r_{j}∥\;<d_{c},\;d_{c}=8.0\;Å.$$

For every admitted edge, the *exact* distance $d_{\mathrm{ij}}$ is stored; it is not rounded to $d_{c}$. Edge lengths range from about 3.8 Å for consecutive backbone pairs to just under 8.0 Å for the most peripheral contacts. The sensitivity of the meaningful temperature window and the contact count to the cutoff $d_{c}$ is examined in Figure S3; the qualitative conclusions remain unchanged for all cutoffs tested. Pairs with $d_{\mathrm{ij}}\geq d_{c}$ are excluded entirely ($w_{\mathrm{ij}}=0$) and play no further role. Both structures remain fully connected at $d_{c}=8.0$ Å, so no residues were discarded.

The resulting networks contain 172 nodes/830 edges (6GOD) and 169 nodes/809 edges (4OBE). The modest size difference reflects the three extra C‐terminal residues (Met170, Ser171, Lys172) resolved in the active structure; these additional nodes introduce not only themselves but also the contacts they form with neighboring residues, accounting for the 21‐edge surplus in 6GOD.

### 2.3 Edge Energies, Boltzmann Weights, and the Role of Temperature

Each edge is assigned an energy proportional to its Cα−Cα distance, which we calibrate via the effective temperature kT

(2) $$\varepsilon_{\mathrm{ij}}=d_{\mathrm{ij}}\;\left[\text{units}:\;Å\right],$$

the simplest monotonic function of distance. Short contacts are energetically favored; peripheral ones are penalized. For 6GOD the range is $\varepsilon_{\min}\approx 3.78$ Å to $\varepsilon_{\max}\approx 7.99$ Å, with a mean of roughly 5.8 Å. Robustness to alternative functional forms (quadratic, inverse, harmonic) was verified but is not reported.

At effective temperature kT (units: Å; see below), each edge receives a Boltzmann weight

(3) $$w_{\mathrm{ij}}\left(\mathrm{kT}\right)=\exp\;\left(-\varepsilon_{\mathrm{ij}}/\mathrm{kT}\right).$$

Since edge energies differ, the weights are heterogeneous at any fixed kT. The ratio between two edges,

(4) $$\frac{w_{\mathrm{ij}}}{w_{\mathrm{kl}}}=\exp\;\left(-\frac{\varepsilon_{\mathrm{ij}}-\varepsilon_{\mathrm{kl}}}{\mathrm{kT}}\right),$$

changes with temperature whenever $\varepsilon_{\mathrm{ij}}\neq \varepsilon_{\mathrm{kl}}.$ The same principle extends to entire spanning trees: given two trees $\tau_{1}$ and $\tau_{2}$ with total energies $E\left(\tau_{1}\right)$ and $E\left(\tau_{2}\right)$ (defined in § 2.5), their probability ratio is

(5) $$\frac{P\left(\tau_{1}\right)}{P\left(\tau_{2}\right)}=\exp\;\left(-\frac{E\left(\tau_{1}\right)-E\left(\tau_{2}\right)}{\mathrm{kT}}\right).$$

Temperature, therefore, acts as a selective force that actively redistributes statistical weight across the spanning‐tree ensemble. At low kT, the partition function is dominated by low‐energy trees, those built from the shortest possible contacts, effectively freezing the system into a high‐affinity backbone. As kT rises, the weight distribution flattens, and the ensemble begins to explore an increasingly diverse set of spanning trees. This redistribution is the physical origin of the observed heat capacity peaks and the kT‐dependent entropy profiles; it confirms that the model captures a genuine thermodynamic ensemble rather than a topologically static description. The cutoff $d_{c}$ enters only once, to fix which edges exist. Temperature never adds or removes edges; it changes only their relative statistical importance within the fixed topology.

As a concrete illustration, at ${\mathrm{kT}}_{\mathrm{ref}}=1.0$ Å a backbone contact (d=3.8 Å) carries weight w≈0.022, whereas a peripheral contact (d=7.9 Å) carries $w\approx 3.7\times 10^{-4}$ roughly two orders of magnitude smaller.

#### 2.3.1 Effective Temperature

The physical contact energy is $\alpha \cdot d_{\mathrm{ij}}$, where α converts angstroms to energy. Defining ${\mathrm{kT}}_{\mathrm{eff}}\equiv k_{B}T/\alpha$ absorbs α into the temperature scale, giving ${\mathrm{kT}}_{\mathrm{eff}}$ units of Å. We write kT for brevity throughout. Because all state comparisons are formed as differences, they are independent of α and carry unambiguous thermodynamic content. Mapping kT onto a Kelvin scale would require calibrating α against experimental observables (e.g., crystallographic B‐factors or NMR order parameters), which is not attempted here.

#### 2.3.2 Physically Sensible Window

Two constraints define a reference window within which the Boltzmann weights most closely approximate the contact hierarchy expected for a well‐folded protein at ambient conditions. The partition function is, however, analytically exact across the full temperature range; thermodynamic features outside this window reflect genuine properties of the network energy landscape, in particular the competition among near‐degenerate low‐energy spanning trees that is inaccessible to single‐minimum descriptions. To bracket the useful temperature range, we impose two constraints on the Boltzmann weights. Core contacts (d≈4 Å) should retain at least 1% of unit weight, ensuring that the shortest interactions remain thermodynamically active; this gives kT>0.87 Å. Peripheral contacts (d≈8 Å) should carry less than 1% of the unit weight, preventing the ensemble from flattening into the topology‐only (GNM) limit; this gives kT<1.74 Å:

(6) 0.87Å<kT<1.74Å.

Within this window, short contacts dominate while multiple spanning trees compete, the regime expected for a well‐folded protein. The reference value ${\mathrm{kT}}_{\mathrm{ref}}=1.0$ Å sits comfortably inside. The full temperature sweep ($\mathrm{kT}\in \left[0.3,6.0\right]$ Å, 60 uniform grid points) extends well beyond this window in both directions, exploring regimes from single‐tree dominance to near‐uniform tree weights.

### 2.4 Weighted Kirchhoff Laplacian

The Boltzmann weights are assembled into an n×n weighted Kirchhoff Laplacian $\Gamma \left(\mathrm{kT}\right)$,

(7) $$\Gamma_{\mathrm{ij}}=\left(\begin{array}{cc} -w_{\mathrm{ij}}, & i\neq j,\;\left(i,j\right)\in e,\\ 0, & i\neq j,\;\left(i,j\right)\notin e,\\ \sum_{k:\left(i,k\right)\in e}w_{\mathrm{ik}}, & i=j,\; \end{array}\right.$$

or equivalently Γ=D−W, where W is the weighted adjacency matrix and D the diagonal matrix of weighted degrees. The matrix is real, symmetric, and positive semidefinite by construction, with a single zero eigenvalue (eigenvector: all‐ones) as long as G is connected. Previous applications of the graph Laplacian to protein networks have focused on individual eigenvalues as structural descriptors [15]; here, the product of all nonzero eigenvalues, the determinant of the reduced Laplacian, gives the partition function exactly.

### 2.5 Spanning‐Tree Partition Function

The sum of the edge energies of a spanning tree is:

(8) $$E\left(\tau \right)=\sum_{e\in \tau }\varepsilon_{e}.$$

It is this tree‐level energy, not any single‐edge quantity.

The weighted Matrix‐Tree Theorem [17, 18] provides three equivalent expressions for the partition function:

(9) $$\begin{array}{c} Z\left(\mathrm{kT}\right)\;=\;\underset{\text{determinant}}{\underbrace{\det\left(\Gamma_{\mathrm{red}}\right)}}\;=\;\underset{\text{product form}}{\underbrace{\sum_{\tau \in \Omega \left(G\right)}\prod_{e\in \tau }w_{e}\left(\mathrm{kT}\right)}}\;\\ \;=\;\underset{\text{Boltzmann form}}{\underbrace{\sum_{\tau \in \Omega \left(G\right)}\exp\;\left(-\frac{E\left(\tau \right)}{\mathrm{kT}}\right)}}. \end{array}$$

The physical mechanism underlying the temperature‐dependent redistribution of statistical weight is illustrated with a minimal four‐node graph in Figure S4, where explicit enumeration of spanning trees clarifies the origin of the heat capacity peak and the entropy rise. Here $\Omega \left(G\right)$ is the set of all spanning trees of G and $\Gamma_{\mathrm{red}}$ is the reduced Laplacian obtained by deleting any one row and the corresponding column, the choice does not affect the result. The first form is what we actually compute: a single determinant replaces the combinatorial sum. The second makes each tree's contribution explicit as a product of edge weights. The third is the canonical statistical‐mechanical form, identifying every spanning tree τ as a microstate weighted by its total energy at inverse temperature $\beta =1/\left(\mathrm{kT}\right)$. It is this identification that turns a graph‐theoretic determinant into a bona fide partition function.

Because G is connected, $\Gamma_{\mathrm{red}}$ is positive definite and $Z\left(\mathrm{kT}\right)>0$ for all kT>0. For numerical stability, we work with the logarithm,

(10) $$\log Z=\sum_{k=1}^{n-1}\log\lambda_{k},$$

where $\lambda_{1},\ldots ,\lambda_{n-1}$ are the eigenvalues of $\Gamma_{\mathrm{red}}$, computed. Only eigenvalues exceeding $10^{-10}$ enter the sum, guarding against numerical zeros.

### 2.6 Thermodynamic Quantities

All thermodynamic observables derive from $\log Z\left(\mathrm{kT}\right)$. Because $Z\left(\mathrm{kT}\right)$ is a finite sum of positive exponentials; it is smooth on $\left(0,\infty \right)$; all quantities below are therefore real‐analytic in kT and possesses derivatives of every order.

#### 2.6.1 Free Energy

See Equation (11).

(11) $$F\left(\mathrm{kT}\right)=-\mathrm{kT}\cdot \log Z\left(\mathrm{kT}\right).$$

#### 2.6.2 Mean Tree Energy

The Boltzmann‐weighted average $\bar{E}\left(\mathrm{kT}\right)$ of $E\left(\tau \right)$ over all spanning trees,

(12) $$\bar{E}\left(\mathrm{kT}\right)=\frac{\sum_{\tau \in \Omega \left(G\right)}E\left(\tau \right)\;e^{-E\left(\tau \right)/\mathrm{kT}}}{\sum_{\tau \in \Omega \left(G\right)}e^{-E\left(\tau \right)/\mathrm{kT}}}={\mathrm{kT}}^{2}\;\frac{d\log Z}{d\left(\mathrm{kT}\right)}.$$

Numerically, we evaluate the right‐hand form by a symmetric finite difference with a relative step $\delta =10^{-4}\cdot \mathrm{kT}$:

(13) $$\bar{E}\left(\mathrm{kT}\right)\approx {\mathrm{kT}}^{2}\cdot \frac{\log Z\left(\mathrm{kT}+\delta \right)-\log Z\left(\mathrm{kT}-\delta \right)}{2\delta }.$$

#### 2.6.3 Entropy

Units of $k_{B}$:

(14) $$S\left(\mathrm{kT}\right)=\log Z+\frac{\bar{E}}{\mathrm{kT}}.$$

#### 2.6.4 Fundamental Relation

These quantities satisfy

(15) $$F=\bar{E}-\mathrm{kT}\cdot S$$

identically an algebraic consequence of Equations ((11), (12), (13), (14)), not an approximation. Verifying that Equation (15) holds to machine precision at every grid point is the single most informative test of the computation.

#### 2.6.5 Heat Capacity

See Equation (16).

(16) $$C_{v}\left(\mathrm{kT}\right)=\frac{d\bar{E}}{d\left(\mathrm{kT}\right)}=\frac{\mathrm{Var}\left(E\right)}{{\left(\mathrm{kT}\right)}^{2}},$$

where $\mathrm{Var}\left(E\right)$ is the variance of $E\left(\tau \right)$ over the Boltzmann ensemble. The second form is the fluctuation–dissipation relation; because a variance cannot be negative, it guarantees $C_{v}\geq 0$ everywhere.

#### 2.6.6 A Note on Absolute Values

Thermodynamic potentials are defined up to an additive constant, so their individual absolute values have no physical meaning. Everything reported in this work is either a state difference $\Delta X=X_{\text{active}}-X_{\text{inactive}}$ or a temperature derivative.

### 2.7 Edge Marginal Inclusion Probabilities

How important is a given contact for holding the network together? The marginal probability that edge $e=\left(i,j\right)$ appears in a randomly sampled spanning tree answers this question:

(17) $$P\left(e\in \tau ;\mathrm{kT}\right)=w_{\mathrm{ij}}\left(\mathrm{kT}\right)\;\cdot \;R_{\mathrm{eff}}\left(i,j;\mathrm{kT}\right),$$

where

(18) $$R_{\mathrm{eff}}\left(i,j;\mathrm{kT}\right)=\Gamma_{\mathrm{ii}}^{†}+\Gamma_{\mathrm{jj}}^{†}-2\Gamma_{\mathrm{ij}}^{†}$$

is the effective resistance between the two nodes in the conductance network ${\left\{w_{e}\right\}}_{e\in E}$, and $\Gamma^{†}$ is the Moore‐Penrose pseudoinverse of Γ [22, 23]. In the GNM, the pseudoinverse element ${\left(\Gamma^{-1}\right)}_{\mathrm{ij}}$ equals the mean‐square fluctuation $\left\langle \Delta R_{\mathrm{ij}}^{2}\right\rangle$ between residues i and j, a quantity termed the dynamic distance [13]. The effective resistance $R_{\mathrm{eff}}\left(i,j\right)$ used here (Equation 18) generalizes this relationship to the weighted Laplacian: in the unweighted limit (kT→∞). The two quantities coincide, establishing a direct correspondence between the network‐theoretic measure of signal propagation and the GNM description of correlated residue motion.

In practice we compute $\Gamma^{†}$ by inverting the reduced Laplacian $\Gamma_{\mathrm{red}}$ (with scipy.linalg.inv) and embedding the result into an n×n matrix whose ground‐node row and the column is zero. All marginal probabilities are clipped to $\left[0,1\right]$ afterwards to absorb floating‐point noise.

A high $P\left(e\in \tau \right)$ means the contact is structurally essential; it shows up in most spanning trees, not merely that the two residues happen to be close. The identity

(19) $$\sum_{e\in E}P\left(e\in \tau \right)=n-1$$

holds by construction (every tree has n−1 edges, so the expected count must be n−1) and serves as a second independent check on the numerics. Inclusion probabilities are reported at ${\mathrm{kT}}_{\mathrm{ref}}=1.0$ Å.

### 2.8 Relationship to the Gaussian Network Model

The present framework and the standard GNM share the same underlying graph G: both connect residues within a cutoff $d_{c}$ and ignore everything beyond it. The difference lies entirely in the edge weights. The GNM sets $w_{\mathrm{ij}}=1$ for every contact; we set $w_{\mathrm{ij}}=\exp\left(-\varepsilon_{\mathrm{ij}}/\mathrm{kT}\right)$.

The GNM is therefore a limiting case. As kT→∞ every Boltzmann weight approaches unity,

(20) $$\lim_{\mathrm{kT}\to \infty }\exp\left(-\varepsilon_{\mathrm{ij}}/\mathrm{kT}\right)=1,$$

and the weighted Laplacian converges to the unweighted one: $\Gamma \left(\mathrm{kT}\right)\to \Gamma^{\mathrm{GNM}}$. The partition function becomes the spanning‐tree count, $Z\to \;|\Omega \left(G\right)|$ is the number of spanning trees, and the thermodynamic quantities collapse accordingly:

(21) $$S\to \log t\left(G\right),\;\bar{E}\to {\bar{E}}_{T},\;C_{v}\to 0,\;F∼-\mathrm{kT}\log t\left(G\right).$$

where ${\bar{E}}_{T}=\;{\left|\Omega \left(G\right)\right|}^{-1}\sum_{\tau }E\left(\tau \right)$ is the unweighted mean tree energy. The entropy saturates at a purely topological invariant; the heat capacity vanishes because all trees are now equiprobable and there is nothing left to discriminate among them. Similarly, the edge inclusion probabilities reduce to the unweighted effective resistances: $P\left(e\in \tau \right)\to R_{\mathrm{eff}}^{\mathrm{GNM}}\left(i,j\right)$.

This limit is more than a theoretical curiosity; it provides an internal consistency check. At high enough kT, every quantity computed from the weighted ensemble must converge to its GNM counterpart; failure to do so would indicate a bug.

To assess the model's ability to capture local structural fluctuations, we compared its predicted B‐factors with those of the original Gaussian Network Model (GNM) and experimental crystal data (see Table S4 and Figure S5). While the spanning‐tree ensemble identifies discrete allosteric channels with high precision, its overall B‐factor agreement remains comparable to the original GNM. This is consistent with the fact that the standard GNM represents the high‐temperature limit of our weighted Kirchhoff framework. Unlike our previous bioinformatic model [13], which explicitly compensates for many‐body crowding effects and solvent exposure to improve B‐factor correlations by 20%–40%, the present model is designed to prioritize the discovery of global thermodynamic landscapes and entropy‐driven switching. Consequently, the deviations from experimental B‐factors often caused by specific chemical structures or local packing densities are not the primary focus of this work. Instead, the model provides the same reliable baseline as the Kirchhoff matrix inversion while introducing a novel capacity to resolve the specific ‘topological highways' of allosteric transmission.

### 2.9 Functional Region Classification

Residues are grouped into functional regions following standard KRAS annotations (Table 1). Inter‐lobe bridge contacts are edges $\left(i,j\right)$ with one endpoint in the effector lobe (i≤86) and the other in the allosteric lobe (j≥87).

**TABLE 1:** KRAS functional region definitions.

Region	Residues
P‐loop	10–17
Switch I	25–40
Switch II	57–75
Effector lobe (other)	1–86 (excl. above)
Allosteric lobe	87–169
C‐terminal	170–172

### 2.10 Numerical Validation

Four checks guard against implementation errors. The absolute thermodynamic quantities for the active and inactive states are shown in Figure S1; they satisfy the relation $F=\bar{E}-\mathrm{TS}$ to numerical precision throughout the entire temperature range.

1. *Fundamental relation*. $|F-\left(\bar{E}-\mathrm{kT}\;S\right)|\;<10^{-12}$ at every grid point (Equation 15).
2. *Marginal sum rule*. $|\sum_{e}P\left(e\in \tau \right)-\left(n-1\right)|\;<10^{-12}$ (Equation 19).
3. *Non‐negative heat capacity*. $C_{v}\geq 0$ everywhere, as guaranteed by the fluctuation–dissipation form (Equation 16).
4. *Moore–Penrose throughout*. The pseudoinverse $\Gamma^{†}$ satisfies

(22) $${\Gamma \Gamma }^{†}\Gamma =\Gamma ,\;\Gamma^{†}{\Gamma \Gamma }^{†}=\Gamma^{†},$$

(23) $${\left({\Gamma \Gamma }^{†}\right)}^{⊤}={\Gamma \Gamma }^{†},\;{\left(\Gamma^{†}\Gamma \right)}^{⊤}=\Gamma^{†}\Gamma .$$

All four passed with residuals below $10^{-12}$ for both structures at every temperature.

### 2.11 Notation

See Table 2.

**TABLE 2:** Symbol definitions used throughout this work.

Symbol	Definition	Equation
*Graph*		
$G=\left(V,e\right)$	Residue contact graph	(1)
n=∣V∣	Number of residues (nodes)	—
∣e∣	Number of contacts (edges)	—
$d_{\mathrm{ij}}$	C*α*–C*α* distance between i,j	(1)
$d_{c}$	Contact cutoff distance	(1)
*Edge‐level energetics*		
$\varepsilon_{\mathrm{ij}}$	Energy of edge $\left(i,j\right)$; equals $d_{\mathrm{ij}}$	(2)
$w_{\mathrm{ij}}\left(\mathrm{kT}\right)$	Boltzmann weight $\exp\left(-\varepsilon_{\mathrm{ij}}/\mathrm{kT}\right)$	(3)
*Spanning trees*		
τ	A single spanning tree of G	(8)
$\Omega \left(G\right)$	Set of all spanning trees of G	(9)
$|\Omega \left(G\right)|$	Number of spanning trees	(21)
$E\left(\tau \right)$	Total energy of tree τ: $\sum_{e\in \tau }\varepsilon_{e}$	(8)
*Laplacian and partition function*		
W	Weighted adjacency matrix	(7)
D	Diagonal degree matrix	(7)
Γ	Weighted Kirchhoff Laplacian D−W	(7)
$\Gamma_{\mathrm{red}}$	Reduced Laplacian (one row/column deleted)	(9)
$\Gamma^{†}$	Moore–Penrose pseudoinverse of Γ	(18)
$\lambda_{k}$	Eigenvalues of $\Gamma_{\mathrm{red}}$	(10)
$Z\left(\mathrm{kT}\right)$	Spanning‐tree partition function	(9)
*Thermodynamic quantities*		
kT	Effective temperature (units: Å)	(3)
β	Inverse temperature $1/\left(\mathrm{kT}\right)$	(9)
F	Free energy −kTlogZ	(11)
$\bar{E}$	Mean tree energy (Boltzmann average)	(12)
S	Entropy $\log Z+\bar{E}/\mathrm{kT}$	(14)
$C_{v}$	Heat capacity $d\bar{E}/d\left(\mathrm{kT}\right)$	(16)
ΔX	State difference $X_{\text{active}}-X_{\text{inactive}}$	(24)
*Edge marginals*		
$R_{\mathrm{eff}}\left(i,j\right)$	Effective resistance between i and j	(18)
$P\left(e\in \tau \right)$	Marginal inclusion probability of edge e	(17)

### 2.12 Parameters

See Table 3.

**TABLE 3:** Complete parameter specification.

Parameter	Symbol	Value	Role
C*α* cutoff	$d_{c}$	8.0 Å	Edge inclusion
Atom type	—	C*α*	Coarse graining
PDB chain	—	A (both)	Chain selection
Energy function	$\varepsilon_{\mathrm{ij}}=d_{\mathrm{ij}}$	Linear in distance	Edge weight $w_{e}=\exp\left(-\varepsilon_{e}/\mathrm{kT}\right)$
Reference temperature	${\mathrm{kT}}_{\mathrm{ref}}$	1.0 Å	Single‐*kT* comparisons
Temperature sweep	kT	$\left[0.3,6.0\right]$ Å	Full thermodynamic profiles
Grid points	—	60 (uniform)	Sweep resolution
Eigensolver	—	scipy.linalg.eigvalsh	Symmetric positive‐definite solver
Eigenvalue floor	—	$10^{-10}$	Guard against numerical zeros
Finite‐diff. step	δ	$10^{-4}\cdot \mathrm{kT}$	Derivative of logZ
Marginal clipping	—	$\left[0,1\right]$	Rounding control
Ground node	—	node 0 (arbitrary)	Reduced Laplacian row/column deletion
Validation tolerance	—	$10^{-12}$	Fundamental relation & marginal sum rule

### 2.13 Implementation

Everything was coded in Python 3 and run inside Google Colaboratory (2‐core CPU, 12 GB RAM). The libraries involved are NumPy (array operations), SciPy (eigendecomposition via scipy.linalg.eigvalsh; inversion via scipy.linalg.inv), NetworkX (graph construction and traversal), and Matplotlib (figures). The full pipeline, PDB parsing through final figures, is deterministic and finishes in under 2 min. No external simulation software is needed.

**FIGURE 1:**
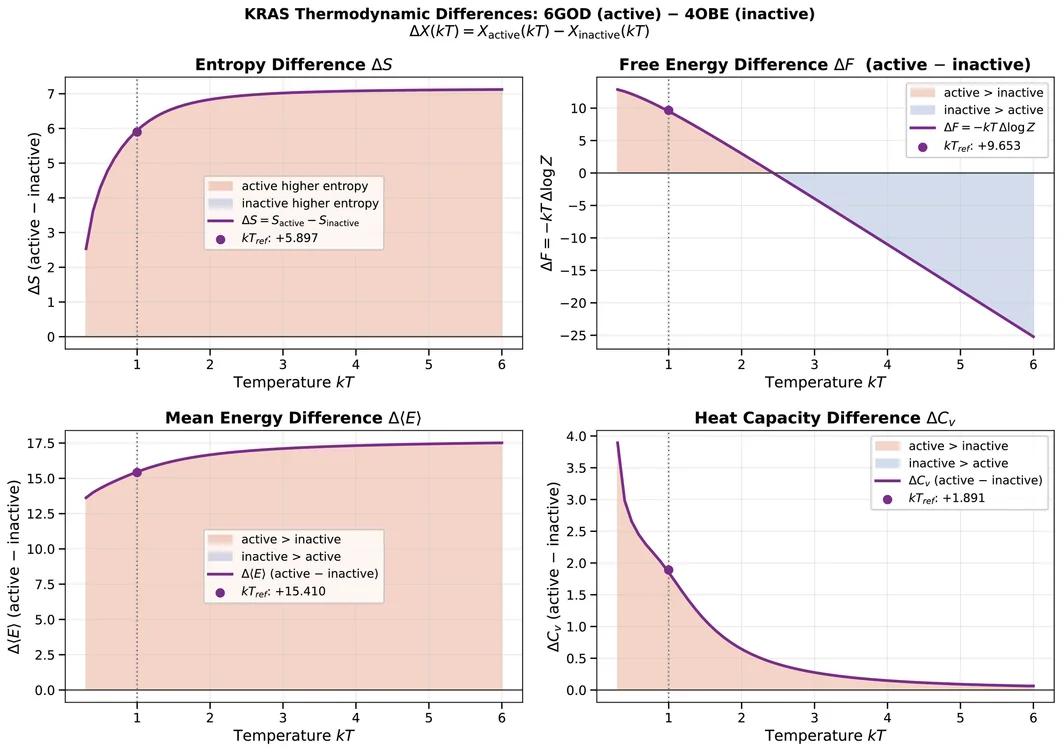
Thermodynamic difference profiles. (${\mathrm{kT}}_{\mathrm{ref}}=1.0$, dotted line). $\Delta X\left(\mathrm{kT}\right)=X_{\text{active}}\left(\mathrm{kT}\right)-X_{\text{inactive}}\left(\mathrm{kT}\right)$ for all four observables across $\mathrm{kT}\in \left[0.3,6.0\right]$. (A) Entropy difference ΔS: Uniformly positive and saturating near ΔS≈7 at high temperature, confirming that the GTP‐bound network supports more spanning‐tree configurations at every kT. At ${\mathrm{kT}}_{\mathrm{ref}}=1.0\;Å$, ΔS=+5.76. (B) Free energy difference ΔF=−kTΔlogZ: Positive at physiological temperatures, crossing zero near kT≈2.4. (C) Mean tree energy difference $\Delta \bar{E}>0$ throughout: The active network pays a persistent energetic cost reflecting the structural reorganization required for effector engagement. (D) Heat capacity difference $\Delta C_{v}$: Large at low temperature and decaying monotonically, indicating greater thermal sensitivity of the active‐state network.

**FIGURE 2:**
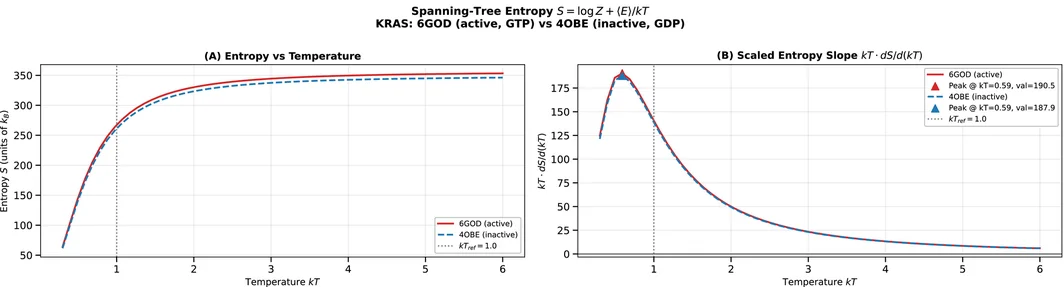
Temperature dependence of spanning‐tree entropy. (A) Absolute entropy [14] (units of $k_{B}$) for 6GOD (red solid) and 4OBE (blue dashed). Both curves increase monotonically and saturate at high temperature, with the active state consistently above the inactive state, indicating higher configurational degeneracy of the GTP‐bound contact network. (B) Scaled entropy slope $\mathrm{kT}\cdot \mathrm{dS}/d\left(\mathrm{kT}\right)$. Both states peak at kT≈0.59Å (6GOD: $190.5\;k_{B}$; 4OBE: $187.9\;k_{B}$), showing that the rate of topological reorganization is maximal, well below ${\mathrm{kT}}_{\mathrm{ref}}=1.0\;Å$, and that the two networks reorganize at nearly identical rates despite their different nucleotide states. The dotted vertical line marks ${\mathrm{kT}}_{\mathrm{ref}}$.

Contact maps (Figures 4 and 5) use imshow with the YlOrRd colormap for absolute probabilities and RdBu_r for differential maps; color limits are clipped to the 95th percentile of absolute values. Thermodynamic profiles (Figures 1 and 2) overlay shaded spans for the functional regions.

## 3 Results

### 3.1 Network Statistics

Table 4 summarizes the basic topological properties of the two contact networks. The active structure 6GOD contains three additional residues and 21 additional contacts relative to 4OBE, reflecting the difference in resolved residues between the two crystals. Despite this size difference, mean and median edge marginal probabilities $P\left(e\in \tau \right)$ are nearly identical (mean 0.206 versus 0.208; median 0.119 in both cases), indicating that the average structural essentiality of a contact is conserved between states. This near‐invariance implies that the two networks are comparably well‐connected and that thermodynamic differences between them arise not from changes in overall connectivity density but from the redistribution of structurally critical contacts across the network.

**TABLE 4:** Contact network statistics for the two KRAS structures at C*α* cutoff 8.0Å.

Property	6GOD (active, GTP)	4OBE (inactive, GDP)
Nucleotide state	GTP‐analog bound	GDP bound
Residues (nodes)	172	169
Contacts (edges)	830	809
Mean $P\left(e\in T\right)$	0.206	0.208
Median $P\left(e\in T\right)$	0.119	0.119

### 3.2 Thermodynamic Difference Profiles

Because individual thermodynamic potentials are defined only up to an additive constant (§ 2.6), the physical content resides entirely in the state differences

(24) $$\Delta X=X_{\text{active}}-X_{\text{inactive}},\;X\in \left\{F,\;\bar{E},\;S,\;C_{v}\right\},$$

which are shown in Figure 1. The free‐energy difference ΔF crosses zero at kT≈2.41Å, a crossover independent of any additive reference constant, marking the effective temperature at which the active and inactive ensembles are thermodynamically balanced. A complete panel of individual quantities, their differences, and the heat capacity is provided in Figure S2, offering a detailed view of the thermodynamic behavior of both states. Below this value, the inactive state is preferred; above it, the active state becomes favored. This balance, and its perturbation by nucleotide binding, underlies the switching mechanism.

### 3.3 Temperature Dependence of Entropy and Heat Capacity

That the heat‐capacity maximum lies below the reference window is structurally expected. The peak reflects the smallest energy scale in the contact network: the energetic cost of substituting one or two edges in the ground‐state spanning tree with slightly longer alternatives, a local intra‐state property of the protein's core packing. This scale is distinct from the interstate switching scale captured by ΔF, ΔS, and $\Delta \bar{E}$, which operates within and above the reference window.

The framework therefore resolves two thermodynamic regimes simultaneously: core contact competition at low kT and global nucleotide‐state discrimination at moderate kT.

Figure 2 shows the thermodynamic entropy.

$$S=\log Z+\frac{\bar{E}}{\mathrm{kT}}$$

for both states as a function of effective temperature. The active state maintains higher entropy across the entire range (ΔS>0), saturating near ΔS≈7 at high kT. This persistent advantage reflects the greater topological degeneracy, that is, the larger number of viable spanning trees of the GTP‐bound network. In the high‐temperature limit, the entropy of each state converges to $\log t\left(G\right)$ (Equation 21), confirming that the saturation value is a topological invariant. The scaled entropy slope $\mathrm{kT}\;dS/d\left(\mathrm{kT}\right)$ (Figure 2B) peaks sharply at low temperature and converges for both states at high temperature, indicating that thermal reorganization of the two networks becomes indistinguishable above kT≈3Å.

The heat capacity

$$C_{v}=\frac{d\bar{E}}{d\left(\mathrm{kT}\right)}$$

reaches a pronounced maximum near kT≈0.53Å, below the physically sensible window of Equation (6) (0.87Å<kT<1.74Å). This feature reflects the activation of the first excited spanning trees above the network's ground state.

At very low temperatures, the Boltzmann weights $w_{\mathrm{ij}}=\exp\left(-d_{\mathrm{ij}}/\mathrm{kT}\right)$ are overwhelmingly dominated by the shortest edges, and the ensemble collapses onto a single dominant tree, the unique connected subgraph composed of the shortest possible edges reaching every residue. As the temperature rises, a small number of slightly longer edges acquire sufficient weight to replace one or two edges in the ground‐state tree, generating a handful of low‐lying excited configurations. The heat capacity peak marks the point of maximal competition between the ground state and these first alternatives. That $C_{v}\to 0$ at high kT (Equation 21) confirms that the peak is a finite‐temperature phenomenon: once all trees are equiprobable, the variance of the tree energy vanishes, and the heat capacity disappears.

**FIGURE 3:**
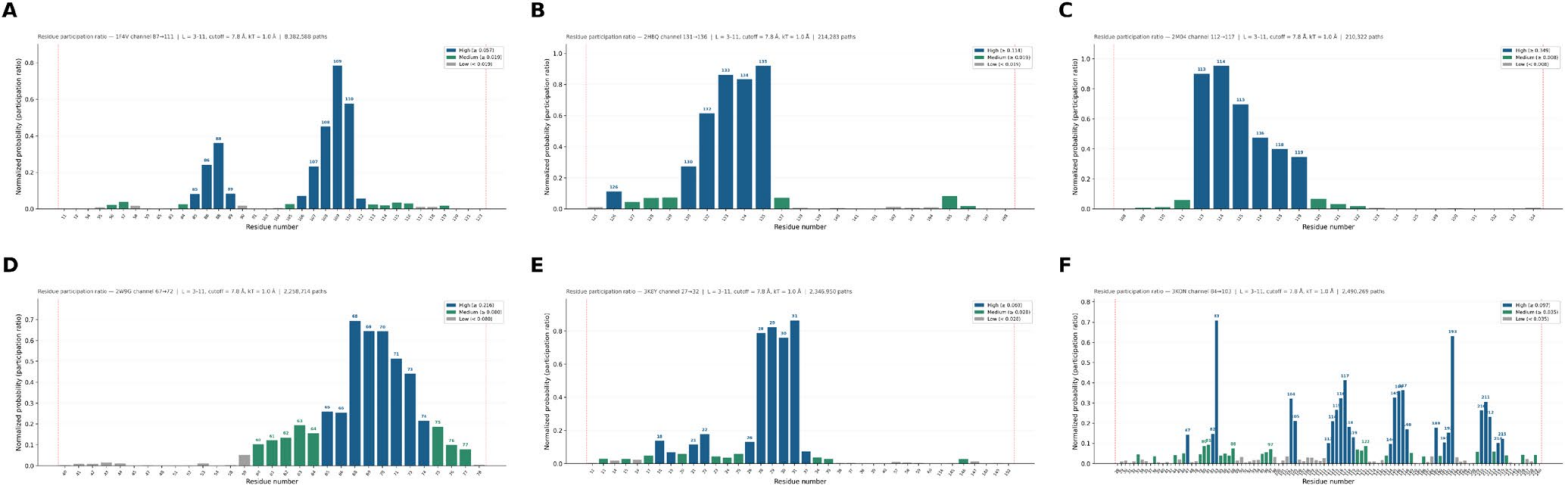
Residue participation probability profiles for six benchmark proteins. Each panel shows the normalized Burton–Pemantle probability of interior residues in the highest‐probability allosteric channel, computed over all simple paths of node length 3≤L≤11. (A) 1F4V, channel 87→111; (B) 2HBQ, 131→136; (C) 2M04, 112→117; (D) 2W9G, 67→72; (E) 3K8Y, 27→32; (F) 3K0N, 84→103. C*α* contact cutoff = 7.8 Å, *k*_*B*_ *T* = 1.0 Å.

That this peak lies below the physically sensible window is natural. It reports on the smallest length scale in the contact network: the difference between the very shortest edges and those that can replace them, typically on the order of 1–2Å. The heat capacity maximum, therefore, reflects local, high‐fidelity core contacts defining the protein's ground‐state architecture, whereas global switching between active and inactive states involves much larger effective energy scales supplied not by temperature but by nucleotide binding (Figure 3).

### 3.4 Edge Inclusion Probabilities

**FIGURE 4:**
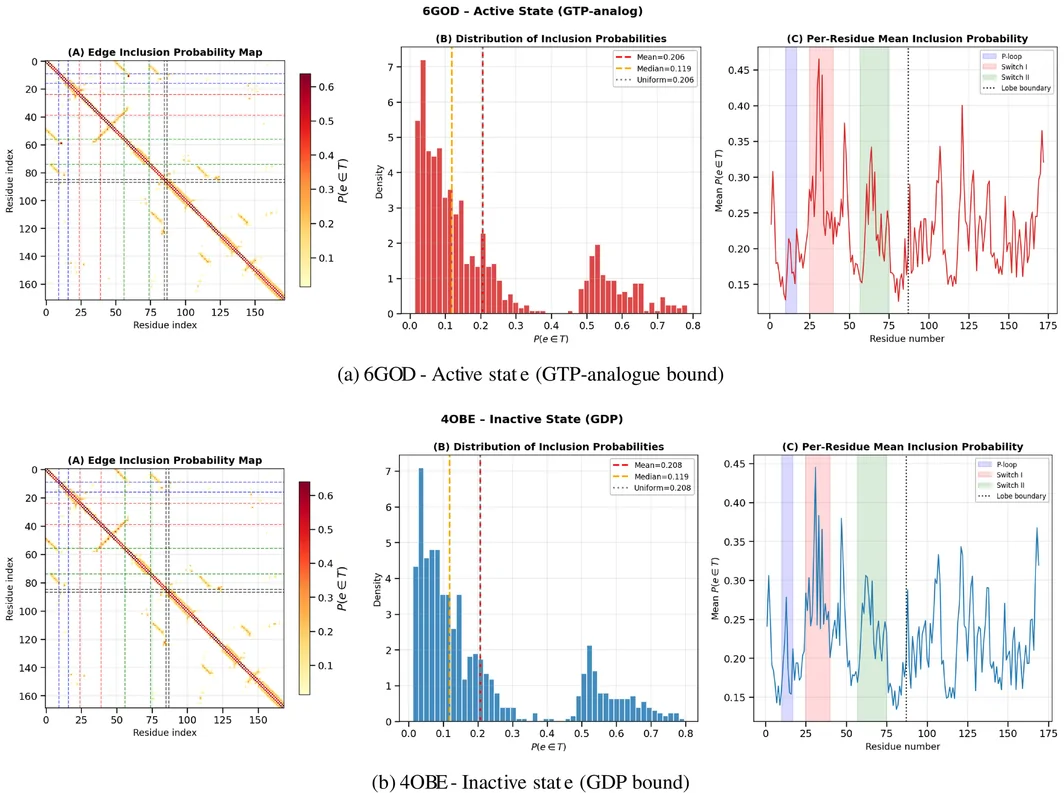
Edge marginal inclusion probability maps. Three‐panel layout for each state. (A) Contact map colored by $P\left(e\in T\right)$ (yellow–red scale, $v_{\min}=0$, $v_{\max}=1$). (B) Histogram of inclusion probabilities with mean (red dashed), median (orange dashed), and uniform reference (gray dotted). (C) Per‐residue mean $P\left(e\in T\right)$ with functional regions shaded: P‐loop (blue, residues 10–17), Switch I (red, 25–40), Switch II (green, 57–75), and lobe boundary (dotted, residue 87).

Marginal edge inclusion probabilities $P\left(e\in T\right)$ at ${\mathrm{kT}}_{\mathrm{ref}}=1.0\;Å$ are shown in Figure 4 for both states. The distributions are broad and right‐skewed, peaking near P≈0.05 with a secondary shoulder near P≈0.5–0.7. Most contacts are topologically redundant; a minority serve as essential backbone edges. The mean P equals the uniform reference $\left(n-1\right)/|E|$, confirming consistency with the sum rule (Equation 19).

This localization is structurally expected. Switch I (residues 25–40) constitutes the direct binding interface for downstream effectors including Raf‐RBD and PI3K*γ*, and its conformational state gates signal transduction to the MAPK and PI3K cascades. That Switch I emerges as the dominant locus of state‐dependent ΔP provides an internal validation of the method, recovering the established primary site of nucleotide‐driven functional reorganization from network topology alone.

### 3.5 Allosteric Communication Pathways Across Diverse Protein Systems

To further check the robustness of the weighted spanning tree model, we calculated residue participation probabilities in the most probable allosteric channels for six systems previously analyzed in our earlier work [24]. The identified topological bottlenecks consistently align with experimentally determined functional sites and communication circuits.

#### 3.5.1 CheY (1F4V)

For the channel 109→114, the residue participation histogram identifies a sharp core backbone relay at residues 110–113, with a maximum at F109. This prediction is in excellent agreement with 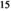
N CPMG relaxation dispersion experiments showing that the F111–E125 network and the $\beta_{4}$–$\alpha_{4}$ loop are integral to the dynamic switching of unphosphorylated CheY [25]. The spanning tree model also captures the participation of the phosphorylation site D57 and its coupled partner T87. These residues appear as distinct, lower‐probability peaks (specifically in the 84–90 tertiary through‐space cluster and near the 57 locus), which is consistent with experimental evidence identifying the D57–T87–Y106 triad as the primary engine for relaying the activation signal to the distal binding surfaces [25, 26].

#### 3.5.2 Cyclophilin A (3K0N)

In cyclophilin A (CypA, PDB 3K0N), NMR relaxation‐dispersion experiments identify an extended coupling network centered on residues R55, H70, and K82 that mediates. Cyclophilin A (CypA) is a peptidyl‐prolyl isomerase whose active site undergoes millisecond‐timescale conformational exchange between minor and major states during catalysis [27, 28]. Subsequent work has shown that the electric field of this network, often referred to as an “electrostatic handle,” helps catalyze proline isomerization by stabilizing the developing charge in the transition state [29]. Signals near catalytic residue S99, including resonances associated with the S99–N102 region, report on vital side‐chain and loop motions that accompany the interconversion between minor and major conformational states. These dynamics are visible in NMR relaxation measurements and are consistent with temperature‐jump and solution‐scattering studies that probe global motions in CypA [30]. Residues around positions 117 and 146 behave as dynamic hubs within flexible loops and helices that exhibit collectivity during the catalytic cycle, even in the absence of substrate. In particular, NMR peaks near residue 117 are associated with the T119–L122 loop segment, a region highlighted by relaxation‐dispersion studies as central to the enzyme's collective motions [31].

Residues such as 193 and 211 are high‐centrality nodes in the inferred allosteric network, extending communication pathways into the peripheral regions of CypA. These sites help coordinate long‐range conformational changes that support efficient peptidyl‐prolyl isomerase activity, as suggested by dynamical‐network and NMR‐informed analyses of the enzyme.

Our model identifies these specific residues as the “topological bottlenecks” that mediate the collective dynamics of CypA. By capturing these validated residues as primary participants in the spanning‐tree ensemble, the model proves its ability to disclose the physical conduits of allosteric transmission in CypA, consistent with our previous findings [24].

#### 3.5.3 HRas (3K8Y)

For the channel 43→48, the model highlights residues 44–45 and 49–51 as primary participants. These high‐probability hubs bridge the GTP‐binding region with the allosteric lobe. This alignment supports the experimental observation that allosteric ligands (such as calcium acetate) disseminated through these regions trigger structural rearrangements in Switch II and the allosteric lobe [32].

#### 3.5.4 DHFR (2W9G)

The allosteric channel 67→72 shows a significant participation peak at residues 68–71. These residues facilitate the communication between the active site and the distal F–G and G–H loops, regions known to be critical for catalytic activity and hydrogen‐bonding stabilization during the enzymatic cycle [33, 34].

#### 3.5.5 Bcl‐xL (2M04)

In the PUMA‐bound state, the channel 112→117 identifies a dominant bottleneck at residues 113–116, with a peak at residue 114. This region acts as a structural conduit bridging the PUMA and p53 binding sites. Our findings coincide with NMR shift data and mutational studies identifying residues in the $\alpha_{4}$ and $\alpha_{5}$ helices as essential for allosteric sensitivity to mitochondrial inhibitors [35, 36].

#### 3.5.6 Caspase‐1 (2HBQ)

The calculated pathway for the channel 131→136 identifies high‐probability peaks at residues 132–135. These residues form a critical hub within the internal allosteric circuit, bridging the active site and distal regulatory regions. While earlier studies have established the salt bridge between R286 and E390 as a primary mediator of allosteric inhibition, our network analysis suggests that the 131–136 segment acts as a significant structural conduit that coordinates these distal movements within the caspase dimer. This suggests an extended, multi‐residue relay that facilitates long‐range allosteric communication across the enzyme [37]. In the Caspase‐1 structure, the region (near the 190 s) is part of a flexible loop/structural element that sits in proximity to the active‐site pocket and is known to be involved in the large‐scale conformational changes required for activation. While they are not classically defined as the “allosteric switch,” they are often implicated in dynamical network analyses as part of the communication relays between the subunits of the Caspase‐1 dimer [38].

### 3.6 Differential Contact Analysis

**FIGURE 5:**
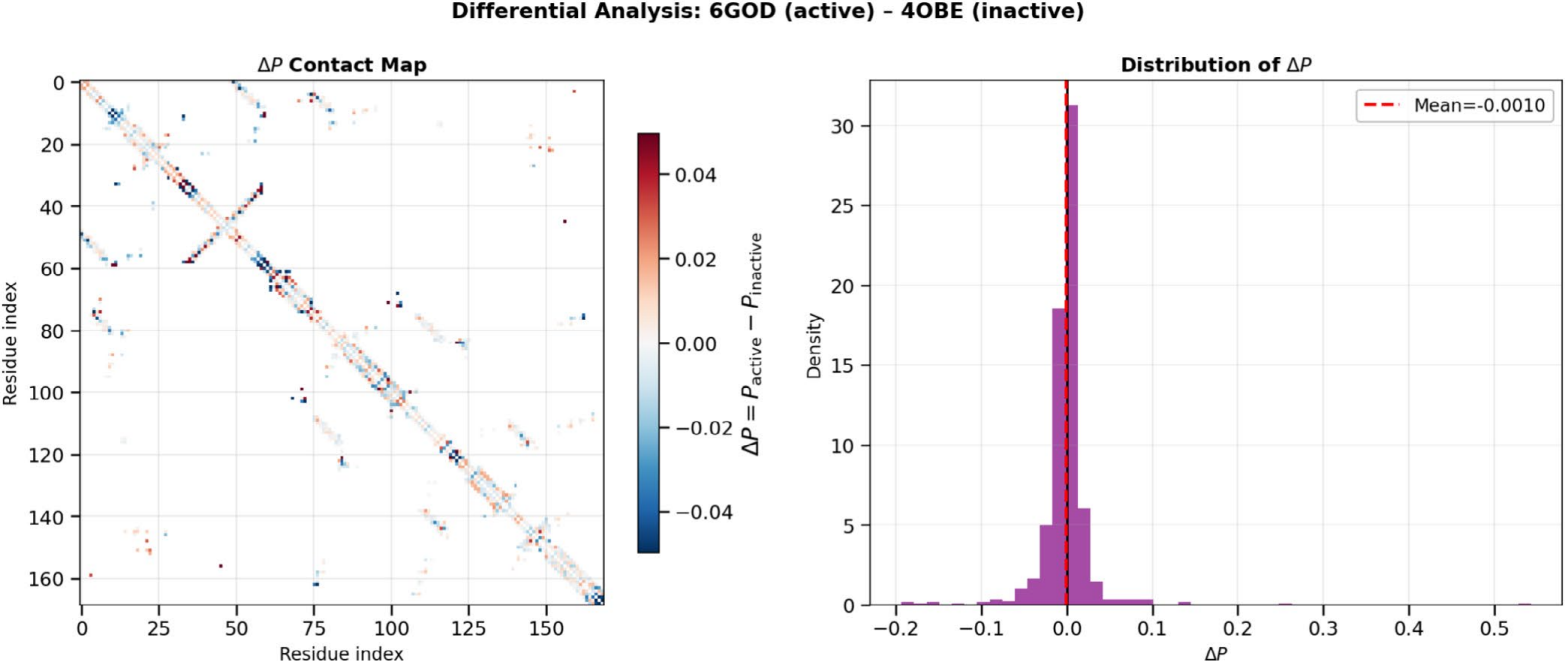
Differential edge inclusion probability analysis. (Left) ΔP contact map (diverging red‐blue color scale; red = active enriched, blue = inactive enriched). Differences are sparse and localized, concentrated in Switch I and at the effector, allosteric lobe interface. (Right) Distribution of ΔP values across 783 common edges; mean = −0.001, confirming marginal conservation of total spanning‐tree weight between states.

Figure 5 presents the differential map $\Delta P=P_{\text{active}}-P_{\text{inactive}}$ for the 783 contacts common to both structures. The mean ΔP=−0.001 is near zero by construction; the informative signal lies in the spatial distribution of deviations. Switch I (residues 25–40) dominates the positive ΔP values (contacts more essential in the active state), followed by the inter‐lobe region near residues 86–90. Negative ΔP contacts, those more essential in the inactive state, concentrate at the Switch II/allosteric lobe interface.

## 4 Principal Findings

The following analysis is based on a single representative crystal structure per nucleotide state. It should therefore be interpreted as a proof‐of‐concept demonstration of the framework rather than a definitive characterization of the KRAS thermodynamic landscape.

The thermodynamic distinction between the active and inactive states of KRAS can be distilled into three quantities: the free energy difference ΔF, the mean tree energy difference $\Delta \bar{E}$, and the conformational entropy difference ΔS. Taken together, they reveal how the protein's contact network is rewired upon nucleotide exchange, and why this rewiring matters.

### 4.1 Free Energy and the Thermodynamic Ground State

At physiological temperatures, the inactive state is thermodynamically favored, carrying lower free energy than the active state (ΔF>0). The GDP‐bound conformation is structurally well integrated: Switch I and Switch II lie collapsed against the protein core, forming a dense, stable network of contacts. Without an external driving force, the system therefore predominantly occupies the inactive basin. GTP binding supplies the free energy required to populate the higher‐free‐energy active conformation.

The *intrinsic* balance point, where neither state is thermodynamically preferred, occurs at kT≈2.41. Unlike the zero‐crossings of the individual free energy curves, which both fall near kT≈2.2 as an artifact of the unit convention (see § 2.6), this crossover is independent of any additive reference. That it lies well above the physiological effective temperature underscores the role of the inactive state as the intrinsic ground state and confirms that activation requires sustained nucleotide‐derived free energy.

### 4.2 Energetic Cost of Activation

Decomposing the free energy difference clarifies its origin. The active state exhibits a consistently higher mean tree energy (ΔE>0): its residue‐residue contacts are, on average, less optimal than those in the inactive state. In the GDP‐bound structure, the switches are held in place by favorable interactions with the core and with each other; GTP binding disrupts many of these, forcing the switches into extended and partially exposed conformations. The resulting energetic cost is not a defect but a necessary investment, the price of creating an accessible effector‐binding surface.

### 4.3 Entropic Gain and Functional Versatility

What justifies this investment is the accompanying gain in conformational entropy. The active state has higher entropy (ΔS>0), indicating that its switch regions are not simply rearranged but access a larger number of competing network configurations. In the inactive state, the switches are confined to a narrow ensemble of low‐energy spanning trees; GTP binding liberates them, granting access to a much broader set of thermally accessible configurations. In network terms, this entropic expansion corresponds to a larger number of topologically distinct spanning trees connecting the nucleotide‐binding pocket to distal surface residues. The formalism establishes this combinatorial diversity directly; its biological correlate, that a richer set of network configurations facilitates engagement of multiple downstream effectors, including RAF, PI3K, and RALGDS through distinct allosteric routes, remains an interpretive hypothesis consistent with the present analysis but requiring validation against conformational and binding data.

### 4.4 Temperature Dependence and the Switching Mechanism

The temperature dependence reinforces this picture. As temperature rises, the entropic term TΔS grows, gradually offsetting the energetic penalty $\Delta \bar{E}$. At sufficiently high effective temperatures, the two states become equally probable (ΔF=0). That the crossover lies well above physiological temperatures reaffirms that the inactive state is the intrinsic ground state and that switching demands sustained nucleotide‐derived free energy.

KRAS activation is therefore not a simple flip from a low‐energy off state to a high‐energy on state. It is a redistribution of thermodynamic resources: the energetic cost of disrupting ground‐state contacts is offset by a gain in conformational entropy, which in turn enables functional versatility. The active state is structurally diverse, and it is this diversity encoded in the network architecture that may enable a single protein to serve as a hub for multiple signaling pathways.

## 5 Discussion

MD simulations by Zhao et al. [6] show that GTP binding enhances conformational flexibility and long‐range interactions in KRAS, consistent with the higher entropy we observe in the active‐state network. Their analysis centers on pairwise correlations and kinetic pathways; our framework quantifies the global network consequences, showing that the entropic gain comes at a persistent energetic cost $\Delta \left\langle E\right\rangle >0$ and that the two states are tuned to operate near a thermodynamic crossover that maximizes switch sensitivity.

Allosteric network modeling of KRAS–effector interactions using Markov state models and mutational scanning reveals that oncogenic mutations redistribute communication pathways between the nucleotide‐binding pocket and the effector interface [9]. The large ΔP we observe in Switch I accords with this picture and provides a direct thermodynamic measure of the redistribution. That a small number of contacts can dominate this process is expected from the small‐world topology of protein contact networks [14], which ensures that a few edges serve as critical bridges for long‐range signal transmission. Edges with high $P\left(e\in \tau \right)$ in our analysis are precisely these bridges: they appear in the majority of spanning trees and constitute the most robust communication channels.

Crystallographic work by Shima et al. [39] established that the GTP‐bound form of Ras exists as an ensemble of two interconverting conformations (states 1 and 2), with the switch regions exhibiting pronounced flexibility, suggesting that conformational entropy plays a key role in activation. Our framework quantifies this entropic contribution directly from network topology.

Pálfy et al. [40] highlighted the critical role of the Mg^2+^‐free state in facilitating nucleotide exchange in oncogenic KRAS mutants, underscoring the importance of conformational flexibility in the switch regions. Our analysis complements their findings: the active state's higher entropy (ΔS=+5.76) reflects the increased conformational freedom of the switches upon GTP binding, a freedom likely coupled to Mg^2+^ release during nucleotide exchange. The underlying molecular mechanism of GTP hydrolysis has been characterized in atomic detail by time‐resolved FTIR spectroscopy [41].

MD studies by Chen et al. [42] align with our finding of higher tree energy in the active state, directly showing that key contacts—particularly between GTP and Switch I weaken or become unstable upon activation. Xu et al. [43] provide complementary structural evidence: although a new interaction forms (Tyr32 with the GTP γ‐phosphate), stable pre‐existing contacts are disrupted. The overall picture supports our conclusion that the active state is characterized by less stable packing and a higher‐energy contact network compared to the tightly coordinated inactive conformation.

Senet et al. [19] showed that Shannon entropy computed over protein subgraphs at graph distance D=1 exhibits a sharp transition near the melting temperature in gpW and varies heterogeneously along the sequence of α‐synuclein. Their local entropy captures the diversity of micro‐environments around each residue; ours captures the combinatorial degeneracy of the entire network. Both derive from the Kirchhoff Laplacian, and both demonstrate that graph‐based entropy can discriminate functionally distinct protein states, at the residue level in their case, at the network level in ours.

The entropic basis of activation bears directly on the inhibitor mechanism. Covalent inhibitors such as sotorasib and adagrasib function by trapping KRAS in the GDP‐bound inactive conformation; within the present framework, this admits a network‐level interpretation: these compounds effectively collapse the spanning‐tree ensemble toward the low‐entropy inactive state, suppressing the combinatorial degeneracy (ΔS>0) that enables engagement of multiple downstream effectors. Whether spanning‐tree multiplicity constitutes a useful metric for prospective inhibitor design remains to be tested against binding data and mutant‐specific structural ensembles.

A limitation of this analysis is its reliance on two static crystallographic structures, which do not capture the full conformational heterogeneity accessible to each nucleotide state. Effects of crystal packing, incomplete loop resolution, and the absence of explicit solvent are not represented. Extending the framework to conformational ensembles derived from molecular dynamics trajectories, or to multiple independently determined crystal structures per state, would provide a more stringent test of these conclusions and constitutes a natural direction for future work.

## 6 Conclusion

We have applied a spanning‐tree partition function analysis to two KRAS crystal structures, revealing thermodynamic differences that are coherent and biologically interpretable. The active (GTP‐bound) state carries a higher energetic cost but a larger conformational entropy, with the intrinsic free‐energy balance crossing at kT≈2.41 Å; Switch I emerges as the primary locus of state‐dependent topological change. These findings support a view of KRAS activation as an entropy‐driven investment: GTP binding pays an energetic price to liberate the switch regions into a diverse ensemble of competing networks configurations.

For KRAS specifically, natural extensions include application to oncogenic mutants (G12C, G12D, G12V), where mutation‐specific shifts in the entropy and enthalpy balance may provide a thermodynamic rationale for differential drug sensitivity, and calibration of the effective temperature scale against molecular dynamics ensembles or experimentally determined B‐factors.

More broadly, the framework requires nothing beyond atomic coordinates and a contact cutoff; it yields exact thermodynamic potentials from a single matrix determinant without sampling, force fields, or normal‐mode approximations. Any protein for which a residue contact graph can be constructed, including multi‐domain signaling hubs, allosteric enzymes, and intrinsically disordered regions upon complex formation, is accessible to the same analysis. The KRAS application presented here demonstrates the approach; the method itself is general.

## Author Contributions

**Fatma Senguler Ciftci:** software, formal analysis, investigation, data curation, visualization, writing – original draft. **Burak Erman:** conceptualization, methodology, supervision, writing – original draft, writing – review and editing.

## Funding

The authors have nothing to report.

## Conflicts of Interest

The authors declare no conflicts of interest.

## Supporting information


**Figure S1:** Individual thermodynamic quantities for active (6GOD) and inactive (4OBE) KRAS networks. (A) Free energy *F* = −*kT* log*Z* vs. effective temperature. Both curves show the expected decrease with temperature. The crossing of zero near *kT*≈2.2 is arbitrary up to a constant, reflecting the point where *Z* = 1 in our units. (B) Mean contact energy $\bar{E}$ versus temperature; the active state (red) lies above the inactive state (blue) at all temperatures. (C) Heat capacity *C*_*v*_ = $d\bar{E}$/*d*(*kT*) vs. temperature; both states show a broad peak near *kT*≈0.7 and converge at high temperature. (D) Entropy *S* = ($\bar{E}$−*F*)/*T* versus temperature; both increase as expected. The consistently higher entropy of the active state reflects its greater conformational flexibility. These plots validate the thermodynamic consistency of the model: *F*, *S*, and $\bar{E}$ obey *F* = $\bar{E}$ − *TS* to numerical precision throughout.
**Figure S2:** Comprehensive nine‐panel comparison of KRAS thermodynamic states. Top row: individual quantities (*F*, $\bar{E}$, *S*) for 6GOD (red)and 4OBE (blue). Middle row: state differences ∆*X* = *X*_active_−*X*_inactive_ for *F*, $\bar{E}$, and *S*. Bottom row: heat capacity *C*_*v*_ for both states (left), heat capacity difference ∆*C*_*v*_ (center), and the scaled entropy slope *kT*·*dS*/*d*(*kT*) (right). Dotted vertical line: *kT*_ref_ = 1.0 Å
**Table S1:** Meaningful *kT* range as a function of contact‐distance cutoff *d*_*c*_. The reference temperature *kT*_ref_ = 1.0 Å used throughout the main text is indicated in the final column.
**Figure S3:** Sensitivity of the reference *kT* range to the contact‐distance cutoff *d*_*c*_. (A) The shaded band spans [*kT*_low_, *kT*_high_] as defined in Equation (2); the dashed line indicates the midpoint *kT*_mid_. Both bounds shift linearly with *d*_*c*_∈{7.0, 7.5, 8.0, 8.5, 9.0}, showing that the qualitative features of the thermodynamic profiles are preserved across this range. (B) Number of Cα–Cα contacts in the KRAS structures (6GOD, active; 4OBE, inactive) as a function of *d*_*c*_. The vertical dotted line marks the main‐text cutoff *d*_*c*_ = 8.0 Å.
**Table S2:** Edge list with distances and character classification.
**Table S3:** Spanning trees with their edge sets and total lengths.
**Figure S4:** Spanning‐tree thermodynamics illustrated on a four‐node toy graph. (a) The contact graph: three backbone edges (*d* = 3.8 Å, solid) and two non‐bonded contacts (*d* = 5.5 and 6.2 Å, dashed). (b) The complete enumeration of three‐edge subgraphs. Eight are connected and acyclic (spanning trees *τ*1–*τ*8), grouped by total energy E(*τ*); two form triangles that leave a node disconnected (crossed out). (c) Boltzmann probabilities of the eight spanning trees at *kT* = 1.0 Å (blue) and *kT* = 3.0 Å (red). At low temperature *τ*1, the all‐backbone path, carries ∼63% of the statistical weight; raising kT redistributes probability toward higher‐energy trees, flattening the distribution. This redistribution is the combinatorial origin of the conformational entropy reported for the full KRAS network. (d) Ensemble entropy *S* = log*Z* + $\bar{E}$/*kT* as a function of effective temperature. The curve rises from near‐zero (single‐tree dominance) toward the equiprobable limit lnt(*G*) = ln8≈2.08 (dotted line). The shaded band marks the physically sensible window (0.87 < *kT* < 1.74 Å), within which short contacts dominate, yet multiple trees compete. Dots mark the two temperatures shown in (c).
**Figure S5:** Comparison of experimental and predicted B‐factors for nine bench mark proteins. Black curves show experimental PDBB‐factors, blue dashed curves show the scaled GNM predictions, and red curves show the scaled spanning‐tree‐weighted Kirch hoff predictions. For each protein, the fitted scale factor *c* and RMSD are reported in the panel legend.
**Table S4:** Summary of B‐factor fitting results. For each protein, cGNM and cST are the optimal linear scale factors obtained by least‐squares fitting to the experimental B‐factors. The improvement is calculated relative to the GNMRMSD.

## Data Availability

All source code used for constructing the residue‐contact graphs, evaluating the spanning‐tree partition functions, and computing the thermodynamic quantities (F, $\bar{E}$, $C_{v}$, and S) is publicly available on GitHub at https://github.com/fatmasenguler/KRAS_spanning_tree. The repository contains the primary Python scripts, Jupyter notebooks, and a detailed README for reproducing the results. The crystallographic structures analyzed in this study (PDB IDs: 6GOD and 4OBE) can be obtained from the RCSB Protein Data Bank (https://www.rcsb.org).
